# Performance of serological tests PGL1 and NDO-LID in the diagnosis of leprosy in a reference Center in Brazil

**DOI:** 10.1186/s12879-018-3653-0

**Published:** 2019-01-07

**Authors:** André Luiz Leturiondo, Ariani Batista Noronha, Monik Oney Oliveira do Nascimento, Cynthia de Oliveira Ferreira, Fabíola da Costa Rodrigues, Milton Ozório Moraes, Carolina Talhari

**Affiliations:** 1 Programa de Pós-Graduação em Medicina Tropical, Universidade do Estado do Amazonas, Manaus, Amazonas Brazil; 2 Fundação Alfredo da Matta, Av Codajás 24, Cachoeirinha, Manaus, Amazonas Brazil; 3 Laboratório de Hanseníase, Instituto Oswaldo Cruz, Fiocruz, Rio de Janeiro, Rio de Janeiro Brazil; 4 Universidade Nilton Lins, Manaus, Amazonas Brazil

**Keywords:** Leprosy, Accuracy, Serologic tests, PGL1, NDO-LID

## Abstract

**Background:**

Early detection of leprosy and multidrug therapy are crucial to achieve zero transmission and zero grade II incapacities goals of World Health Organization. Leprosy is difficult to diagnose because clinical forms vary and there are no gold standard methods to guide clinicians. The serological rapid tests aid the clinical diagnosis and are available for field use. They are easy to perform, do not require special equipment or refrigeration and are cheaper than the molecular tests.

**Methods:**

**W**e evaluated the performance of two rapid serological tests (PGL1 and NDO-LID) in the discrimination of leprosy cases from healthy individuals at the Alfredo da Matta Foundation, a reference center for the disease in Manaus, Amazonas, Brazil. PGL1 and NDO-LID rapid tests are capable of detecting specific antibodies of *M. leprae*, IgM and IgM/IgG, respectively. A total of 530 healthy subjects and 171 patients (50 with paucibacillary and 121 multibacillary leprosy) were included in the study.

**Results:**

Among the paucibacillary leprosy patients, the sensitivity was 34.0 and 32.0% for the NDO-LID and PGL1, respectively. In multibacillary leprosy patients, the NDO-LID sensitivity was 73.6% and the PGL1 was 81.0%. Serological tests demonstrated specificities of 75.9% for PGL-1 and 81.7% for NDO-LID. The positive predictive value (PPV), negative predictive value (NPV) and accuracy in multibacillary patients were 47.9, 93.1, and 80.2% respectively for the NDO-LID, and 43.4, 94.6 76.8% for PGL1.

**Conclusions:**

The tests showed limited capacity in the diagnosis of the disease, however**,** the high negative predictive value of the tests indicates a greater chance of true negatives in this group favoring exclusion of leprosy. This characteristic of the ML flow test is important in aiding clinical Diagnosis, especially in a region endemic to the disease and with other confounding skin conditions.

**Electronic supplementary material:**

The online version of this article (10.1186/s12879-018-3653-0) contains supplementary material, which is available to authorized users.

## Background

Leprosy is chronic infectious diseases that show a long incubation period. The disease is caused by *Mycobacterium leprae* or *M. lepromatosis* that affect the skin and peripheral nerves. If not early detected and adequately treated, the disease may lead to physical incapacities and irreversible deformities [1]. The introduction of multidrug therapy (MDT), in the early 1980s, had a huge impact in the prevalence of leprosy; more than 16 million patients were cured in the last 30 years [2]. However, the incidence is still high where approximately 200,000 new cases are diagnosed each year. Probably, MDT has a modest impact on incidence because transmission occurs prior to diagnosis. Recent strategies to stop leprosy transmission rely on prophylactic protocols using rifampicin and/or BCG [3, 4]. The World Health Organization (WHO) developed a strategy to achieve zero transmission, zero cases among children with grade II incapacities by 2020 [5]. In Brazil, there are still difficulties in achieving this goal, due not only to the lack of professionals experienced in diagnosing leprosy, but also to the inherent issues of the disease. Some paucibacillary (PB) and neural forms may be easily confused with other common dermatoses such as granuloma annulare, sarcoidosis or pityriasis alba [6]. In addition, about 30% of patients, many of them multibacillary (MB), do not present cardinal symptoms such as loss of sensitivity, favoring the active transmission of the disease [7].

The development of a simple and practical test, able to confirm the clinical decision, is of vital importance, especially in the field, where there are few specialists to diagnose and treat the disease. Slit skin smear and histopathological examinations, despite high specificity, have low sensitivity [8]; those techniques are also invasive and require trained professionals. Molecular techniques such as PCR and qPCR are promising because of their high accuracy, however, these tools are still costly and require skilled technicians [9]. Serological tests, although not stand-alone diagnosis tools, are point-of-care in the early identification of leprosy, even before the initial lesions appearance [10, 11]. Moreover, these tests present a lower cost when compared to molecular assays, are of easy execution, suitable for field diagnosis and require no special equipment or refrigeration [12, 13].

However, the sensitivity of the serological tests varies, depending on the population studied, in order to have a real specific profile of each locality [14]. In this context, this study analyzed the performance of two rapid serological tests for identification of patients with leprosy, at a REFERENCE Center for the disease in the north of Brazil. The two tests exhibited a high negative predictive value (NPV), useful to exclude leprosy supporting clinical diagnosis in endemic regions.

## Subjects, materials and methods

### Design and study population

Evaluation of two serological tests, immunochromatographic, with untreated leprosy patients and healthy individuals who attended by spontaneous demand at the Alfredo da Matta Foundation (Manaus-AM, Brazil). All included patients and controls were 10 years-old or older and were recruited, from March 2014 to March 2016. Patients with leprosy were classified according to the clinical, slit skin smears and histopathological findings [15] and were treated as paucibacillary or multibacillary, according to the number of skin lesions, slit skin smear result and the compromised nerve [16]. The endemic control group was among individuals who lived in the same endemic area as the cases. These endemic controls seek a medical certificate for employment purposes. These individuals were subjected to a dermatoneurological examination and had no suspected leprosy lesions and declared no information concerning contact with leprosy or tuberculosis patients. The study did not include volunteers on corticosteroid treatment, cancer chemotherapy or HIV. Samples that presented hemolyzed serum were also excluded.

This study was approved by the Research Ethical Committee (555.620–13/03/2013) of the ¨Alfredo da Matta Foundation¨. All participants signed an informed consent before enrolment. Participants under the age of 16 have had the consent provided by a parent or legal guardian.

### Detection by NDO-LID and PGL1

The NDO-LID (Orange Life, Rio de Janeiro, Brazil) rapid test uses as antigens a semi-synthetic disaccharide (ND) attached to the octyl (O) radical, which mimics PGL1, conjugated with two fusion proteins, ML 0304 and ML 0331, forming the LID, capable of recognizing, respectively, IgM and IgG antibodies against *M. leprae*. The antigen immobilized on the nitrocellulose membrane, from the PGL1 rapid test (IPTSP/UFG), was the native phenol-1 glycolipid, which detects specific IgM antibodies. Rapid tests followed the instructions recommended by the manufacturer. Two independent technicians performed reading to avoid inconsistencies. A third reader gave his final opinion on discordant results.

### Statistical analysis

For the calculation of sensitivity, specificity, positive predictive value, negative predictive value, with 95% confidence level, MedCalc statistical software for Windows, version 17.0.4 (MedCalc Software, Ostend, Belgium; https://www.medcalc.org; 2016) was used. To evaluate the level of concordance of the trials we used Kappa concordance test (κ) [17]: low (0–0.5), moderate (0.51–0.75) and excellent (0.76–1). The chi-square test was used to verify the association between batches, biological samples and the frequencies of the rapid test result and the clinical-laboratory variables. *P*-value < 0.05 was considered statistically significant.

## Results

A total of 530 healthy subjects and 250 leprosy patients, ranging in age from 10 to 77 years-old (mean of 41.24 years-old) were enrolled in the study. The group most affected by the disease was between 21 to 60 years-old (64.8%), mostly men (66.0%), mixed (85.0%) and had finished elementary school (47%) (Table 1).

**Table 1:** Distribution of leprosy cases according to socio-demographic variables

Characteristics	Number	Percentage (%)
Gender		
Male	165	66.0%
Female	85	34.0%
Total	250	100.0%
Age group (years)		
10–20	44	17.6%
21–40	78	31.2%
41–60	84	33.6%
> 60	44	17.6%
Total	250	100.0%
Education		
Illiterate	11	6.1%
Elementary School	85	47.0%
High school	56	30.9%
Higher education	15	8.3%
Ignored	14	7.7%
Total	181	100.0%
Ethnicity^a^		
Caucasian	9	6.5%
African	8	5.7%
Asian	3	2.1
Mixed	119	85.0%
Indigenous	1	0.7%
Total	140	100.0%

^a^self-reported Ethnicity

Out of 250 patients with leprosy, 250 blood and 171 serum samples were collected. First, we compared the performance of the test NDO-LID between biological samples from serum and blood of the same patient and between two different commercial batches (L-1 and L-2). Thirty samples (13 PB and 17 MB) were randomly selected to verify the sensitivity of the rapid test. Among the 13 PB patients tested, we detected 2 (15.4%) in L-1 and 1 (7.7%) in L-2 when whole blood samples were probed. The serum from the same patients was tested and 6 (46.2%) in L-1 and 2 (15.4%) in L-2 were detected, demonstrating better sensitivity for serum as compared to blood. Among the 17 MB patients, positivity was higher, as expected: 9 (52.9%) and 8 (47.1%) for L-1 and L-2 batches, respectively. While in serum, results were far more sensitive and 14 (82.4%) in L-1 and 11 (64.7%) in L-2 (data not shown).

Then, 171 patients (50 PB and 121 MB) were tested for PGL1 and NDO-LID in serum since results suggested that this sample was the best. The specificity was evaluated in 530 healthy volunteers. The Kappa value was used to compare the agreement rates between samples in both tests. Paucibacillary patients exhibited the excellent concordance and demonstrating that there was no significant difference between them. For MB leprosy patients and healthy controls, the tests had an excellent agreement, with significant differences between them. PGL1 was superior to NDO-LID in detecting MB patients. However, this test also detected a higher number of healthy volunteers indicating lower specificity (Table 2).

**Table 2:** Agreement between NDO-LID and PGL-I rapid tests in serum

	NDO-LID+PGL1-(%)	NDO-LID-PGL1+(%)	AGR (%)	K (IC)	*p*-value*
**MB**	0.0%	7.4%	92.6%	O.79 (0.66 - 0.92)	0.0024
**PB**	2.0%	0.0%	98.0%	0.95 (0.87 - 1.04)	0.8324
**EC**	0.0%	5.8%	94.2%	0.83 (0.77 - 0.89)	< 0.0001

*MB* multibacillary leprosy patients, *PB* paucibacillary leprosy patients, *EC* endemic control, *AGR* agreement,

*k* kappa value, + = positive; − = negative. *Chi-square test

Among the PB leprosy patients, sensitivity was 34.0 and 32.0% for NDO-LID and PGL1, respectively (Table 3). In MB patients, NDO-LID sensitivity was 73.6% and PGL1 was 81.0% (Table 4). Among healthy individuals, specificity was 81.7 and 75.9% for NDO-LID and PGL1 respectively, indicating that both tests have a high percentage of false positives. The positive predictive value (PPV), negative predictive value (NPV), and accuracy in MB leprosy patients were 47.9, 93.1 and 80.2%, respectively for the NDO-LID, and 43.4, 94.6, and 76.8%, for PGL1 (Table 4).

**Table 3:** ML Flow performance test in paucibacillary leprosy patients and healthy volunteers

ML Flow	Sensitivity	Specificity	PPV	NPV	Accuracy
NDO-LID	34% (17/50)	81.7% (433/530)	14.9%	92.9%	77.6%
PGL1	32% (16/50)	75.9%(402/530)	11.1%	92.2%	72.1%

*PPV* Positive Predictive Value, *NPV* Negative Predictive Value

**Table 4:** ML Flow performance test in multibacillary leprosy patients and healthy volunteers

ML Flow	Sensitivity	Specificity	PPV	NPV	Accuracy
NDO-LID	73.6% (89/121)	81.7% (433/530)	47.9%	93.1%	80.2%
PGL1	81.0% (98/121)	75.9% (402/530)	43.4%	94.6%	76.8%

*PPV* Positive Predictive Value, NPV Negative Predictive Value

We decided to test clinical-laboratory parameters evaluating only NDO-LID, because this test exhibited the highest accuracy. One may observe that there was a significant difference in the rapid test for MB leprosy patients with positive skin smear, lepromatous lepromatous leprosy and with more than five lesions (*p* <  0.05) (Additional file 1: Table S1).

## Discussion

Although we know the limited capacity of serological tests in the diagnosis of leprosy, they are still important as a tool to aid clinical diagnosis. This study allowed evaluating the performance of two tests in a region of high endemicity for leprosy and with that to draw a better alternative to their use in the clinical practice. NDO-LID and PGL1 tests may be useful as a support tool for clinical diagnosis. Furthermore, they could be employed for excluding leprosy as a possible cause of a skin lesion, especially in endemic areas where other common dermatological conditions are detected.

When we assessed the best biological samples to be used in the study, no statistical association was found between them or between batches (data not shown), despite the better serum sensitivity than blood. A similar result was also found in other studies, showing a strong correlation between the results from whole blood and serum [13, 18].

Although the evaluation of the tests presented excellent agreement, they did not have the same performance in the identification of patients with leprosy. Both tests had a better capacity to detect individuals with MB leprosy, but were inefficient for the diagnosis of PB forms of leprosy, thus confirming that serological tests could be considered effective tools for the diagnosis of MB leprosy [12, 14, 19, 20]. However, we found a lower sensitivity for MB forms of the disease than the previously reported. In other studies, the sensitivity of NDO-LID antigens in MB patients ranged from 87.0 to 95% [14, 19-22]. This trend has been also observed among leprosy patients classified according to the WHO operational classification, both for rapid tests using immunochromatography, and for those using ELISA methodology. The seropositivity found in the tests under analysis reflects the type of immune response developed by the host [14, 19, 20, 22, 23]. Regarding specificity, other studies found values higher than ours, of 88 and 96.1% for NDO-LID [19, 22]. This might probably indicate differences in the endemicity in the regions where the studies were performed [24]. In assessing the specificity of the rapid tests, we did not include other confounding dermatoses with leprosy, such as granuloma annulare, sarcoidosis, pityriasis alba or mycobacterial infections like tuberculosis, for example.

Both NDO-LID and PGL1 tests showed very low capacity to detect true positives in PB patients (14.9 and 11.1%, respectively) and in MB patients (47.9 and 43.4%, respectively). In fact, 93.1% of NPV is high demonstrating that a negative NDO-LID test could be employed in excluding leprosy as a possible cause of a skin patch or lesion.

Nevertheless, a positive result in the rapid test cannot be used to include patients since we found several false positive samples (20%) among controls. Our control group is composed of healthy individuals living in a high endemicity region. Thus, the elevated seropositivity in this group suggest that this population is regularly being exposed to *M. leprae*, and likely to suggest active transmission [25]. We suggest that ML flow tests (NDO-LID or PGL1) represent an important test as an indicator of *M. leprae* circulation for the surveillance evaluating whether it can be considered an active transmission area. Recently, in a household contact cohort in Bangladesh a positive anti-PGL-I was not a good predictive marker of leprosy outcome [26]. Here, our results of positive ML flow among healthy individuals are far more difficult to predict whether it could be used to estimate the risk of progression towards leprosy since anti-PGL-I could be a surrogate markers of infection. After the evaluation, all positive healthy individuals (*n* = 128) were contacted by telephone 3 years after serological testing and only one responded. In this preliminary analysis, we observed that the adherence is very low, and unfortunately follow up is difficult to achieve in a group of healthy individuals.

It is not yet possible to use any molecular, genetic or serological marker in the diagnosis or prognosis of leprosy. Infection can be demonstrated by PCR or ML Flow and ELISA (PGL-I) serological tests, but these tests are not able to predict, who will progress towards the disease among household contacts. Currently, the diagnosis of leprosy is still based on the appearance and recognition of clinical signs and symptoms. But, improvement of DNA-based detection of *M. leprae* or host targets by qPCR could be useful tools to aid clinical diagnosis.

Only the involvement of experienced physicians, government, population of hyperendemic area [27], along with novel technologies such as qPCR to diagnose [28] and large-scale public policies clinically screening household contacts with chemo- and immunoprophylaxis of healthy ones could halt *M. leprae* transmission in the community and reduce the number of cases of visible disability [29].

## Conclusions

The tests showed limited capacity in the diagnosis of the disease. However, a negative result from the LID-1 or NDO-LID tests showed a greater probability of ruling out the diagnosis of leprosy as a possible cause of a skin lesion. This ML flow test feature is important, especially in an endemic region of the disease and other confusing skin conditions.

## Additional files

Additional file 1: **Table S1.** Evaluation of NDO-LID performance in patients with leprosy. (DOC 36 kb)

Additional file 2: Raw database. Clinical, laboratory and demographic variables used in this study. (XLS 226 kb)

## Abbreviations

AMF: Alfredo da Matta Foundation
ELISA: Enzyme-linked immunosorbent assay
IgG: Immunoglobulin G
IgM: Immunoglobulin M
IPTSP/UFG: Instituto de Patologia Tropical e Saúde Pública/ Universidade Federal de Goiás
MB: Multibacillary
MDT: Multidrug therapy
ML flow: Measurement lateral flow test
NDO-LID: Natural disaccharide octyl – leprosy IDRI diagnostic 1
NPV: Negative predictive value
PB: Paucibacillary
PCR: Polymerase chain reaction
PGL1: Phenolic glycolipid 1
PPV: Positive predictive value
qPCR: quantitative polymerase chain reaction
