# Supplementary material for: Performance of serological tests PGL1 and NDO-LID in the diagnosis of leprosy in a reference Center in Brazil
Source: BMC Infect Dis. 2019 Jan 7;19:22. doi: 10.1186/s12879-018-3653-0 (PMC6322275; doi:10.1186/s12879-018-3653-0)
Supplement: Supplementary file 1 — Table S1. Evaluation of NDO-LID performance in patients with leprosy. (DOC 36 kb) [file 12879_2018_3653_MOESM1_ESM.doc]

Supplementary Table 1

Evaluation of NDO-LID performance in patients with leprosy

| Clinical-laboratory parameters | NDO-LID | |  |
| --- | --- | --- | --- |
|  | Pos | Neg | p value* |
| Operational Classification  Paucibacilary (PB)  Multibacillary(MB) | 17 (34%)  89 (73.6%) | 33 (66%)  32 (26.4%) | 0.0015 |
| Total | 106 | 65 |  |
| Clinical Form  I  TT  BT  BB  BL  LL | 6 (27.3%)  17 (40.5%)  2 (66.7%)  44(68.8%)  3 (100%)  33 (91.7%) | 16 (72.7%)  25 (59.5%)  1 (33.3)  20 (31.2%)  0 (0%)  3 (8.3%) | 0.0001 |
| Total | 105 | 65 |  |
| Skin Smear  Negative  Positive | 48 (47.1%)  56 (93.3%) | 54 (52.9%)  4 (6.7%) | 0.0001 |
| Total | 104 | 58 |  |
| Bacilloscopic Index (BI)  0,00  0,25 a 2  2,01 a 4  4,01 a 6 | 48(47.1%)  11(78.6%)  21 (95.5%)  22 (100%) | 54(52.9%)  3 (21.4%)  1 (4.5%)  0 (0%) | 0.0002 |
| Total | 102 | 58 |  |
| Number of lesions  0  1 a 5  >5 | 5 (45.5%)  29 (42.0%)  46 (86.8%) | 6 (54.5%)  40 (58.0%)  7 (13.2%) | 0.0215 |
| Total | 80 | 53 |  |

*Chi-square test, Confidence Interval (CI) of 95%
